# Supplementary material for: Edge superconductivity in multilayer WTe2 Josephson junction
Source: Natl Sci Rev. 2020 May 30;7(9):1468–75. doi: 10.1093/nsr/nwaa114 (PMC8288511; doi:10.1093/nsr/nwaa114)
Supplement: nwaa114_Supplemental_File [file nwaa114_supplemental_file.pdf]

Supplementary Information for

Edge superconductivity in Multilayer WTe_2 Josephson junction

Ce Huang[1,2†], Awadhesh Narayan[3†], Enze Zhang[1,2], Xiaoyi Xie[1,2], Linfeng Ai[1,2], Shanshan Liu[1,2], Changjiang Yi[4], Youguo Shi[4,5], Stefano Sanvito[6], Faxian Xiu[1,2,7*]

[1] State Key Laboratory of Surface Physics and Department of Physics, Fudan University, Shanghai 200433, China
[2] Collaborative Innovation Center of Advanced Microstructures, Nanjing 210093, China
[3] SSCU, Indian Institute of Science, Bengaluru 560012, India
[4] Institute of Physics and Beijing National Laboratory for Condensed Matter Physics, Chinese Academy of Sciences, Beijing 100190, China
[5] School of Physical Sciences, University of Chinese Academy of Sciences, Beijing 100190, China
[6] School of Physics, AMBER and CRANN Institute, Trinity College, Dublin 2, Ireland
[7] Institute for Nanoelectronic Devices and Quantum Computing, Fudan University, Shanghai 200433, China

[†]These authors contributed equally to this work.

[*]Correspondence and requests for materials should be addressed to F. X. (E-mail: Faxian@fudan.edu.cn).

Content:

I. Device fabrication and characterizations
II. Parameters of all junctions
III. Josephson junction properties in other samples
IV. Josephson junction in different regimes
V. Analysis of the current density profile
VI. Fits of the critical current-magnetic field relation
VII. Additional devices
VIII. Discussion on non-uniform supercurrent density
IX. References

I. Device fabrication and characterizations

WTe$_2$ crystals were mechanically exfoliated onto SiO$_2$/Si substrates. Supplementary Figure 1a-b shows a typical cleaved sample and fabricated device (#5, 40 nm-thick WTe$_2$). The thickness of WTe$_2$ in this study is varied from 10 nm to 60 nm. The Josephson junctions are fabricated using standard *e*-beam lithography as mentioned in Methods and the process is shown in Supplementary Fig. 1c-f. The SEM image of the junction is shown in Supplementary Fig. 1g.

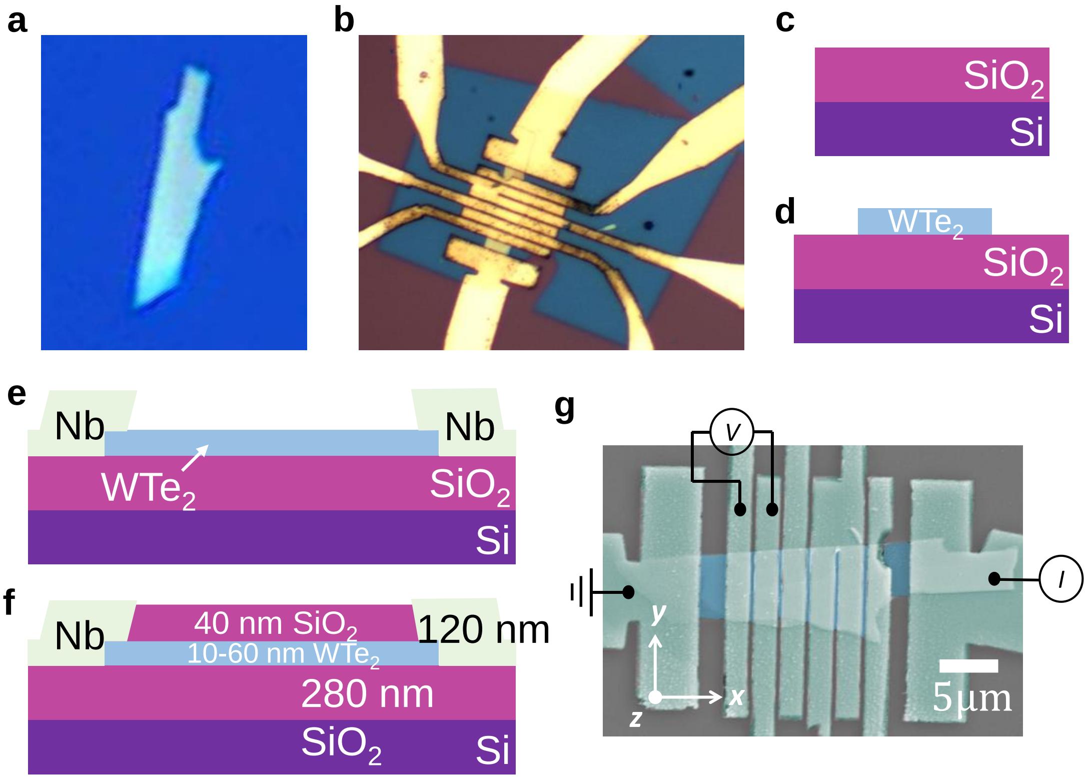

Supplementary Figure 1. The fabrication process of multilayer WTe$_2$ Josephson junctions. (a) An optical picture of 40 nm-thick exfoliated WTe$_2$ (device #5). **(b)** Optical image of the Josephson junction device. A SiO$_2$ protecting layer (blue color) is deposited on the top of the WTe$_2$ flakes. **(c)-(f)** Schematics of a WTe$_2$ Josephson junction fabrication process. Firstly, the WTe$_2$ flakes are mechanically exfoliated from the bulk crystal onto a SiO$_2$/Si substrate. 120 nm-thick Nb electrodes are deposited. 40 nm-thick SiO$_2$ is then deposited to prevent WTe$_2$ from the oxidization. **(g)** Colorized SEM picture highlighting the WTe$_2$ flake (in blue) and the Nb electrodes (in green).

The thickness of WTe$_2$ flakes can be reliably determined by atomic force microscopy (AFM). As an example, Figure S2a-b shows AFM image of device #5 with the clearly-defined step-edges. Furthermore, we measured the thickness along the two edges to ensure the homogeneity of samples as displayed in Supplementary Fig. 2c. Cross-sectional plots in Supplementary Fig. 2d demonstrate that the thickness fluctuation is less than 1 nm. Since the current density can be affected by the roughness at each position along the *y*-direction, the AFM results confirm the uniform roughness in the channel.

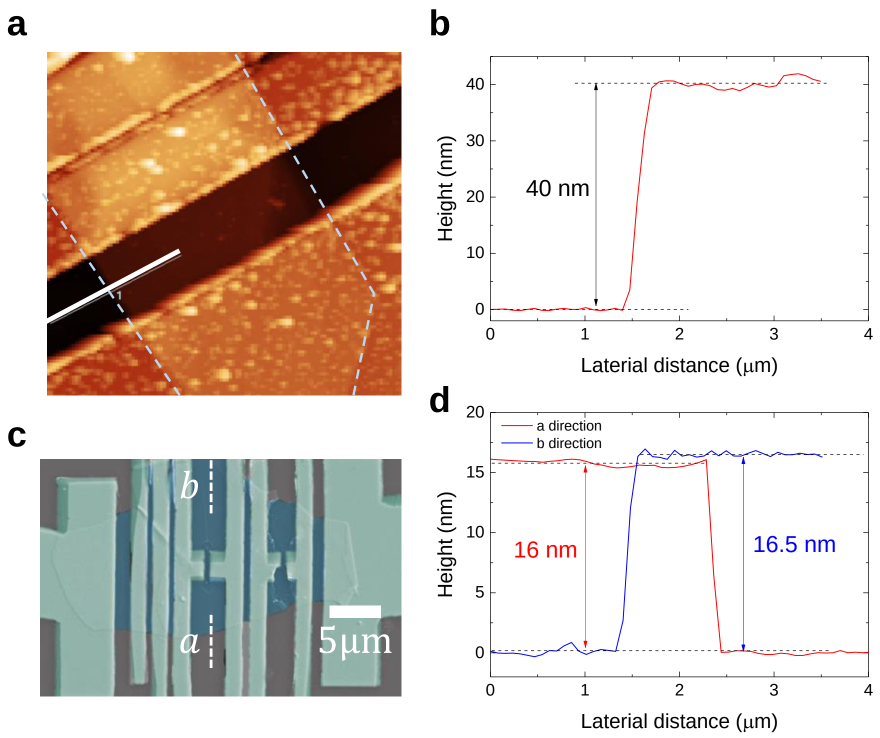

Supplementary Figure 2. AFM images of two WTe_2 Josephson junctions. (a) AFM image of device #5. **(b)** AFM measurements of device #5 as Cross-sectional plots along the white line shown in **(a)**. **(c)** SEM image of device #3. **(d)** Cross-sectional plots along the white dashed lines in (c) (*a* and *b* directions).

II. Parameters of all junctions

Table S1. Parameters of all junctions.

Device	t (nm)	L (nm)	W (μm)	R_N (Ω)	I_c (μA)	T_c (K)	Δ_i (μeV)	ξ_N (nm)
**#1**	10	200	13	1.8	4.1	0.72	108	480
**#2**	13	340	9	1.0	5.0	0.80	120	440
**#3**	16	410	13	1.5	2.0	0.60	90	580
**#4**	20	250	13	2.3	3.0	0.9	135	390
**#5**	40	300	6	0.35	19	0.92	138	300
**#6**	40	350	6.5	2.3	1.5	0.58	87	480
**#7**	60	375	4.5	0.61	6.8	1.1	165	250
**#8**	60	240	6	0.35	20	0.80	120	340
**#9**	12	200	10	2.8	6.0	1.6	240	220
**#10**	50	250	5	0.4	17	1.1	165	320

The thickness $\boldsymbol{t}$ is measured by AFM. The length $\boldsymbol{L}$ and width $\boldsymbol{W}$ are obtained through SEM and Optical microscopy. The normal resistance $\boldsymbol{R_N}$, critical current $\boldsymbol{I_c}$, transition temperature $\boldsymbol{T_c}$ can be extracted from the *R-T* and *I-V* curves. The superconducting gap and coherence length are estimated by $\boldsymbol{\Delta_i = 1.76 k_B T_c}$ and $\xi_N = \frac{\hbar v_F}{\pi \Delta}$, respectively.

III. Josephson junction properties in other samples

Temperature dependences of four typical junctions' resistance are shown in Supplementary Fig. 3. We can identify two transition temperatures in all 4 devices: T_{c1}~8 K is for the Nb superconducting transition temperature; and T_{c2} varies from 0.5 K to 1 K for the Josephson effect.

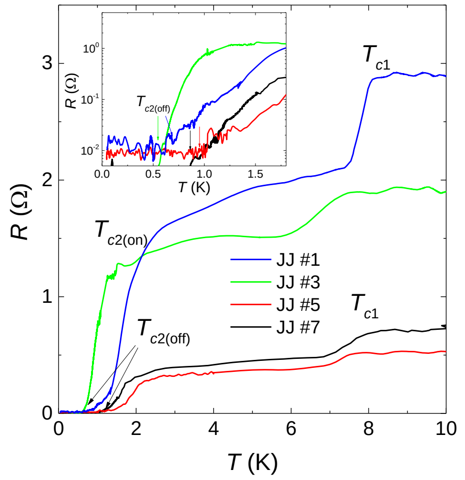

Supplementary Figure 3. Temperature dependence of resistance for devices #1, 3, 5, and 7. The inset shows the close-up in the $T_{c2(off)}$ region.

IV. Josephson junction in different regimes

We choose two typical devices (#1 and #8) to discuss their characterizations here. The Fermi velocity v_F, mobility μ and Fermi vector k_F are evaluated from the Hall effect and Shubnikov–de Haas (SdH) quantum oscillation measurements performed before[1]. The extracted mobility is $\mu = 1.16\ \mathrm{m}^2 \cdot V^{-1} \cdot s^{-1}$ and $\mu = 0.26\ \mathrm{m}^2 \cdot V^{-1} \cdot s^{-1}$ for thick and thin WTe_2, respectively. The Fermi vector is $k_F = 0.052 \text{Å}^{-1}$ and $k_F = 0.057 \text{Å}^{-1}$ for thick and thin WTe_2, respectively. The Fermi velocity v_F is 2.0×10^5 m/s and 2.5×10^5 m/s for the thick and thin WTe_2, respectively.

Then, the superconducting coherence length ξ_N is estimated to 340 nm and 480 nm for device # 8 and #1, respectively, which is close to the junction length L. However, the virtual effective length is longer considering the London penetration depth[2] λ_L~100 nm for Nb such that $L_{eff} = L + 2\lambda_L$, which can be confirmed by the Fraunhofer pattern in the SQI measurements. Then, $\xi_N < L_{eff}$ and it is in long junction regime which gives the relation of $I_c R_n \propto 1/L$.

Meanwhile, we also extract a mean free path $l_{MFP} = 1.3\ \mu\text{m}$ and $0.15\ \mu\text{m}$ for thick and thin WTe_2, respectively. In thick WTe_2, the mean free path is larger than the junction length $l_{MFP} > L$, suggesting that the junctions are in the ballistic regime. In contrast, the thin WTe_2 is in the diffusive regime. To further confirm the ballistic or the diffusive behavior of the junctions, the critical current as a function of temperature, $I_c(T)$, was measured for devices #1 and #8.

For thick WTe_2 device #8 which is a long ballistic junction, the critical current follows with[3]

$$I_c \propto \exp(-k_B T/\delta E)$$

where $\delta E \approx \hbar v_F / 2\pi L_{eff}$ is expected to be independent of carrier density or mobility (as long as the junction remains ballistic). I_c is plotted on a semi-logarithmic scale and clearly shows exponential dependence at high-temperature T as shown in Supplementary Fig. 4a. The little deviation at low temperature is common which has been observed in graphene long ballistic junction[3]. The energy scale δE can be extracted by the slope of linear fitting. Consequently the effective length $L_{eff} = 0.62$ µm is smaller than mean free path $l_{MFP} = 1.3$ µm which accords with the ballistic condition.

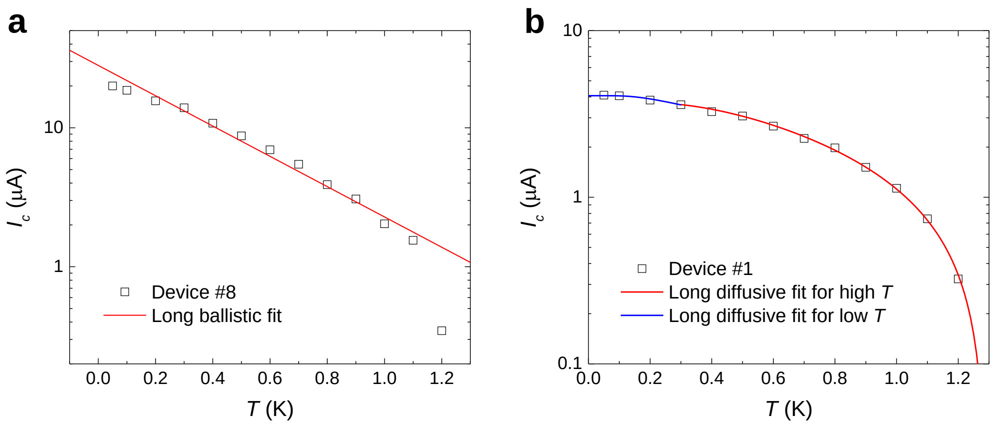

Supplementary Figure 4. Temperature dependence of the critical current. (a) Temperature dependence of the critical current for device #8 fitted by the long ballistic model. The I_c is plotted on a semi-log scale and the red line shows the linear fit. **(b)** Temperature-dependent critical current I_c of device #01 for the long diffusive condition. The red and blue curves are fitted in high and low-temperature regime, respectively.

For thin WTe_2 device #1 which belongs to a long diffusive limit, we used another model to fit as shown in Supplementary Fig. 4b. The model is divided into two temperature regimes. The high-temperature satisfies $k_B T \gg E_{Th}$ (or Δ), where $E_{Th} = \hbar D/L^2$ represents the Thouless energy. E_{Th} is estimated to 8 meV in device #1 and consequently $T > 0.1$ K. Combined with the Usadel equations, the critical current is[4]

$$eR_N I_c \propto k_B T \sum_{n=0}^{\infty} \frac{L}{L_{\omega_n}} \frac{\Delta^2 \exp\left(-L/L_{\omega_n}\right)}{\left[\omega_n + \Omega_n + \sqrt{2(\Omega_n^2 + \omega_n \Omega_n)}\right]^2}$$

where R_N is the normal metal resistance, $\omega_n = (2n+1)\pi k_B T$ is the Matsubara frequency, $\Omega_n = \sqrt{\Delta^2 + \omega_n^2}$, $\Delta \approx \Delta_0 \sqrt{1 - \left(\frac{T}{T_c}\right)^2}$ is the temperature-dependent superconducting gap and $L_{\omega_n} = \sqrt{\hbar D / 2\omega_n}$. As shown in the red curve, the fitted long diffusive model is in excellent agreement with the experimental data at high temperatures. In low-temperature regime, the numerical solution can be approximated by[4]

$$R_n I_c \propto a(1 - be^{-a/3.2 k_B T})$$

where a and b are coefficients. As shown by the blue curve in Supplementary Fig. 4b,

the equation fits the data well. Therefore, our thin WTe_2 satisfies the long diffusive junction.

V. Analysis of the current density profile

In a Josephson junction immersed in a magnetic field B ($B \perp I$), the magnitude of the maximum critical current $I_c^{max}(B)$ depends strongly on the supercurrent density between the Nb electrodes. Here, we convert our measured interference patterns to their originating supercurrent density profiles by the method developed by Dynes and Fulton[5].

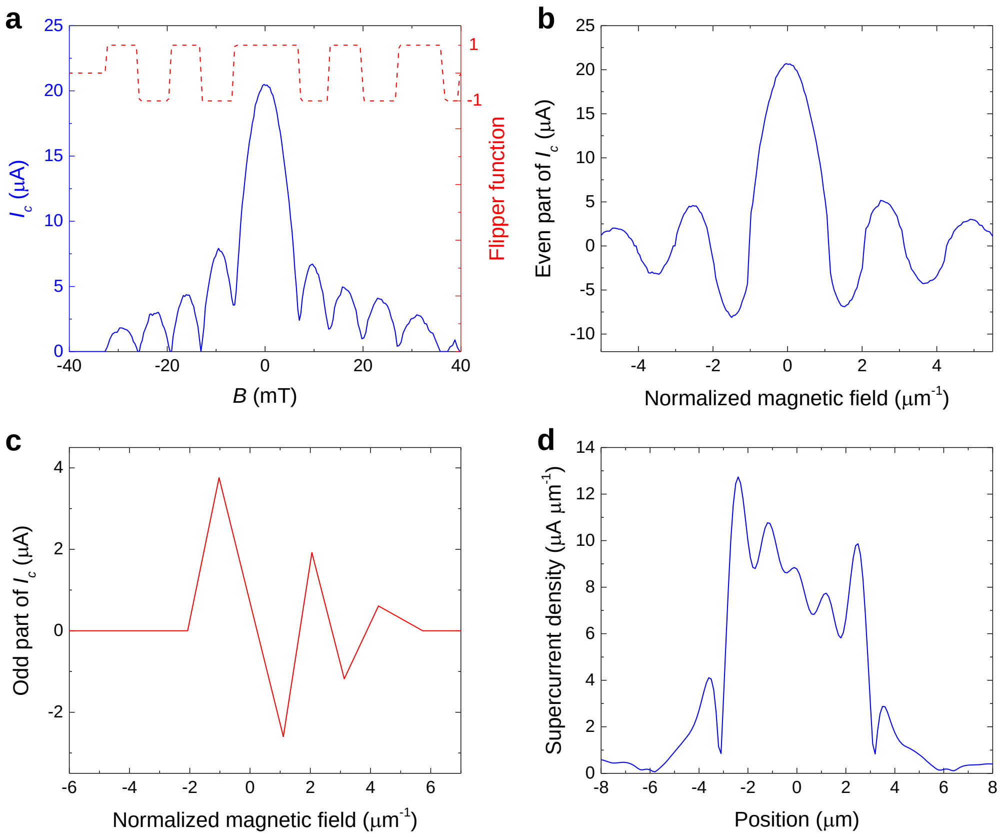

Supplementary Figure 5. Analysis of current density profile in device #8. (a) Recovering the critical current phase. When the current distribution is mostly symmetric, the experimentally observed critical current envelope (blue line) approaches zero between peaks. In such cases, a flipping function (red dashed line) that changes sign at each node of the envelope enables the recovery of $J_C(B)$ from $I_c^{max}(B)$. **(b)** The recovered critical current $I_E(\beta)$, corresponding to the even part of the current density profile $J_E(z)$. **(c)** The recovered critical current $I_O(\beta)$, corresponding to the odd part of the current density profile $J_O(z)$. **(d)** The current density profile $J_S(z)$.

At a fixed magnetic field, supposed the supercurrent density profile $J_S(z)$, its complex Fourier transform yields a complex critical current function $J_C(\beta)$

$$J_C(\beta) = \int_{-\infty}^{\infty} J_S(x)\, e^{i\beta x} \mathrm{d}x$$

where the normalized magnetic field unit $\beta = \frac{2\pi L_{eff} B}{\Phi_0}$. The experimentally observed

$I_C^{max}(B)$ is the magnitude of this summation: $I_C^{max}(B) = |J_C(\beta)|$. We use the even ($I_E(\beta)$) and odd part ($I_O(\beta)$) extracted from the $I_C^{max}(B)$. Then the $J_C(\beta)$ can be expressed as

$$J_C(\beta) = I_E(\beta) + iI_O(\beta)$$

The observed critical current $I_C^{max}(B) = \sqrt{I_E^2(\beta) + I_O^2(\beta)}$ is therefore dominated by $I_E(\beta)$ except at its minimum points. Approximately, $I_E(\beta)$ is obtained by multiplying $I_C^{max}(\beta)$ by a flipping function that switches sign between adjacent lobes of the envelope function (Supplementary Fig. 5a-b). When $I_E(\beta)$ is minimal, the odd part $I_O(\beta)$ dominates the critical current. $I_O(\beta)$ can then be approximated by interpolating between the minima of $I_C^{max}(\beta)$, and flipping sign between lobes (Supplementary Fig. 5c). A Fourier transform of the resulting complex $J_C(\beta)$, over the sampling range b of β, yields the current density profile (Supplementary Fig. 5d):

$$J_S(z) = \left| \frac{1}{2\pi} \int_{-b/2}^{b/2} J_C(\beta) e^{-i\beta z} \mathrm{d}\beta \right|$$

VI. Fits of the critical current-magnetic field relation

Since the Josephson current density is non-uniform (edge-stepped nonuniform) by edge modes, the model of the nonuniform supercurrent density provides a much better fit to the data than that of a uniform supercurrent density. Here we consider the normalized supercurrent density is J_e and 1 in edge and bulk, respectively. This produces an edge-stepped nonuniform supercurrent density $J_c(y)$ along the y direction, as schematically shown in Supplementary Fig. 6a and is given by

$$J_c(y) = \begin{cases} J_e, & 0 \le y \le s, W - s \le y \le W \\ 1, & s < y < W - s \end{cases}$$

where s and W are the edge thickness and junction width. In general, I_c can be expressed as[6]

$$I_c(B) = \left| \int_0^W J_c(y) e^{iky} \mathrm{d}y \right|$$

where $k = 2\pi L_{eff} B/\Phi_0$. Combining the above two equations, the magnetic field dependence of I_c is given as[6]

$$\frac{I_c(B)}{I_c(0)} \simeq \frac{\left(\frac{1}{J_e}\right)\left(\frac{W}{s}-2\right)}{\left(\frac{1}{J_e}\right)\left(\frac{W}{s}-2\right)+2} \times \left| \frac{\sin\left(\frac{\pi B}{B_0}\right)}{\frac{\pi B}{B_0}} + 2J_e\left(\frac{s}{W}\right) \frac{\sin\left[\left(\frac{s}{W}\right)\left(\frac{\pi B}{B_0}\right)\right]}{\left(\frac{s}{W}\right)\left(\frac{\pi B}{B_0}\right)} \cos\left[\left(1-\frac{s}{W}\right)\frac{\pi B}{B_0}\right] \right|.$$

Here, B_0 is the width of each lobe. At the high magnetic field, the value $\frac{\pi B}{B_0}$ increases sufficiently beyond 1 and the second cosinusoidal term pre-dominates, which represents the typical $\cos\left(\frac{\pi B}{B_0}\right)$-type B modulation of I_c similar to a SQUID pattern. In device #2, $B_0 = 0.21$ mT and $W = 9$ µm. Since the supercurrent density at the two sides may be different, we use the model to fit the negative and positive magnetic field data as shown in Supplementary Fig. 6b-c. The B-field modeulation of critical

current agrees well with this model both in its magnitude and field periodicity. The length of the edge is fitted as $s{\sim}0.70$ and $1.65\ \mu\text{m}$ at two sides and the average value is $1.18\ \mu\text{m}$ and close to the Fourier imaging method in Fig. 2g. The edge/bulk supercurrent density ratio is 3.0 and 3.6 at two sides. The good fit of Supplementary Fig. 6b-c indicates the edge-dominant supercurrent and provides clear confirmation of edge supercurrent by edge channels in few-layer WTe_2 with a low bulk supercurrent density.

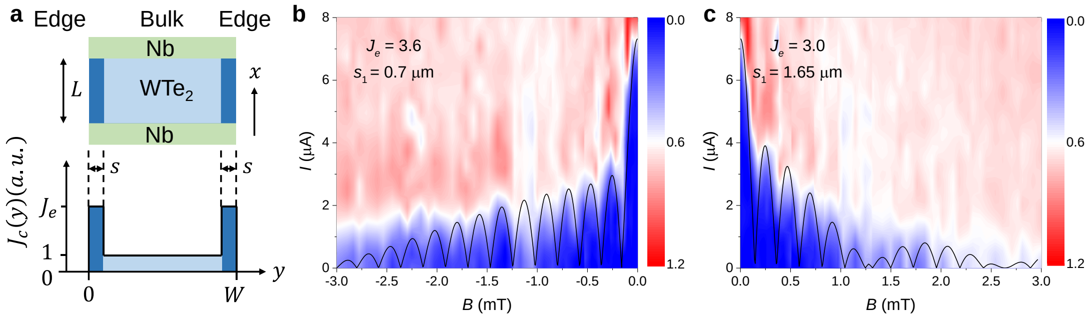

Supplementary Figure 6. Fits of the critical current-magnetic field relation $I_c - B$ for device #2. (a) A schematic cross-sectional view of superconducting WTe_2. Lower panel is the distribution of the supercurrent density $J_c(y)$ along the y axis. s is the thickness of the edge-dominant region. L and W are the length and width of the WTe_2 flake, respectively. **(b)-(c)** Fits of the critical current at positive and negative magnetic field region, respectively.

VII. Additional devices

We also reproduce the Fraunhofer and SQUID pattern transition in the other three WTe_2 devices (#5, #3 and #1) as shown in Supplementary Fig. 7.

The R_2 (device #3) does not oscillate with the magnetic field as shown in Supplementary Fig. S8. On the contrary, with a small magnetic field 0.25 T, the superconducting behavior disappears. The center lobe (black line) can be simulated by Fraunhofer pattern as

$$I_c(B) = I_{c0}\left|\sin\left(\frac{\pi L_{eff}BW}{\Phi_0}\right) / \left(\frac{\pi L_{eff}BW}{\Phi_0}\right)\right|$$

We can estimate the width W_2 for R_2 is about 1.9 μm, which corresponds well with the actual junction width $1.7\ \mu\text{m}$ as shown in Fig. 3a.

Even the second lobe is hard to be distinguished in R_2. We think it is related to the incomplete superconductivity (Josephson effect) in WTe_2. The resistance decrease in R_2 is only smaller than 10% indicating that the proximity Josephson effect just starts and a very small magnetic field can quench the superconductivity in WTe_2.

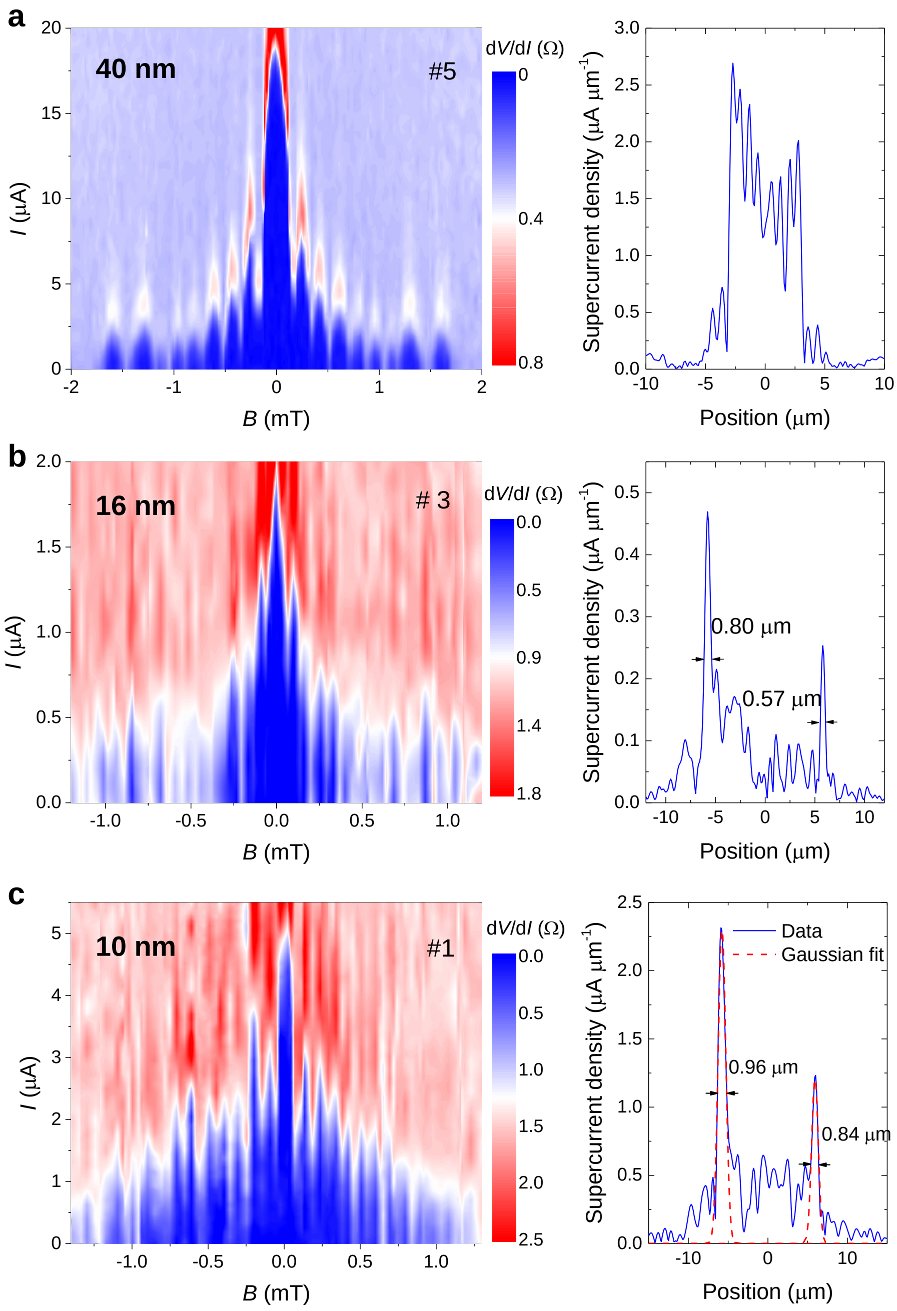

Supplementary Figure 7. Reproducible edge superconductivity. (a) Patterns and current density profiles in a 40 nm-thick WTe_2 (device #5), showing a Fraunhofer pattern with uniform supercurrent density. The white scale bar is equal to 10 μm. **(b)-(c)** SQI results in 16 and 10 nm-thick WTe_2 (device #3 and 1, respectively) indicating edge superconductivity. The widths of the supercurrent-carrying edge channels in **(c)** are estimated using a Gaussian line shape as depicted in the red dashed line.

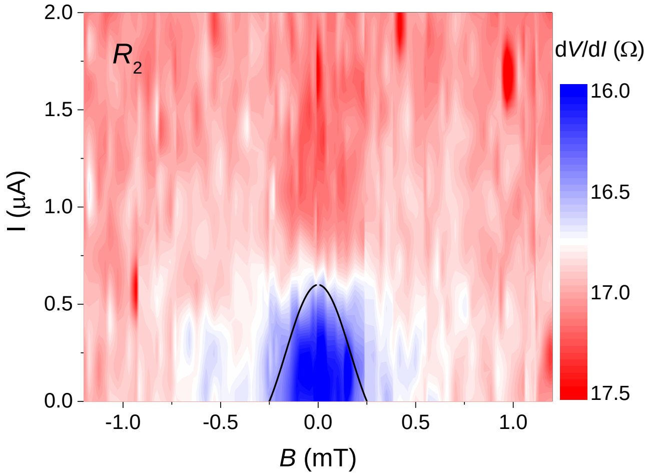

Supplementary Figure 8. SQI results in R_2 (16 nm-thick, device #3). The black line shows the simulation result of the central lobe from the Fraunhofer pattern.

We also performed the control experiment on a similar device structure as sample # 3 by two-probe configuration is shown in Supplementary Fig. 9a. Sample #9 is made by different channels as shown in Supplementary Fig. 9a. R_1 and R_2 represent the Josephson channel across edge or not, respectively. The width of the R_1 and R_2 are $10\ \mu\text{m}$ and $7\ \mu\text{m}$, respectively. Both channels' lengths are about $200\ \text{nm}$ which is smaller than device #3 in Fig. 3. The shorter and wider channel of R_2 helps to increase the possibility of bulk superconductivity. We observed the Josephson effect in both channels as shown in Supplementary Fig. 9b. The critical current is $I_{c1} = 6.0\ \mu\text{A}$ and $I_{c2} = 0.67\ \mu\text{A}$ for R_1 and R_2, respectively and indicates the huge reduction of I_{c2} because of the elimination of edge superconductivity in R_2. We obtain a period of $\sim 0.25\ \text{mT}$ for R_1, which yields the effective length of $L_{eff} = \Phi_0/(\delta B_{lobe} W) \sim 0.8\ \mu\text{m}$. The central lobe length is about $\sim 0.3\ \text{mT}$ which is just a little larger and two times smaller than the I_c oscillation period. Therefore, we can regard the pattern of R_1 as a mixture of Fraunhofer and SQUID-like while traditional Fraunhofer pattern in R_2 in Supplementary Fig. 9c-d. The supercurrent distribution by the inverse Fourier transform illustrates edge and uniform superconductivity for R_1 and R_2, respectively as shown in Fig. R9e. The corresponding supercurrent distribution is highly edge-like with small bulk density and bulk-dominated for R_1 and R_2, respectively. The R_1 widths of the supercurrent-carrying edge channels are estimated to be $1.2 - 1.3\ \mu\text{m}$. On the contrary, the R_2 shows no edge superconductivity because of the lack of edge channels. Furthermore, the asymmetric critical currents at different current directions as shown in Supplementary Fig. 9f which indicates a non-symmetric behavior $I_c^+(B) \neq I_c^-(B)$, where $+$ and $-$ denote the sweep direction of the bias current and $+B$ and $-B$ are the magnetic field directions.

The two-probe configuration method on thick WTe_2 (#10) is shown in Supplementary Fig. 10a. We briefly summarize the results next. Different from the edge superconductivity in #9, the thick WTe_2 exhibits the traditional Fraunhofer pattern (Supplementary Fig. 10b), uniform supercurrent density (Supplementary Fig. 10c) and

symmetry critical current pattern (Supplementary Fig. 10d). The effective length is estimated to be L_{eff}~1.0 μm which is also larger than the channel length L~0.25 μm.

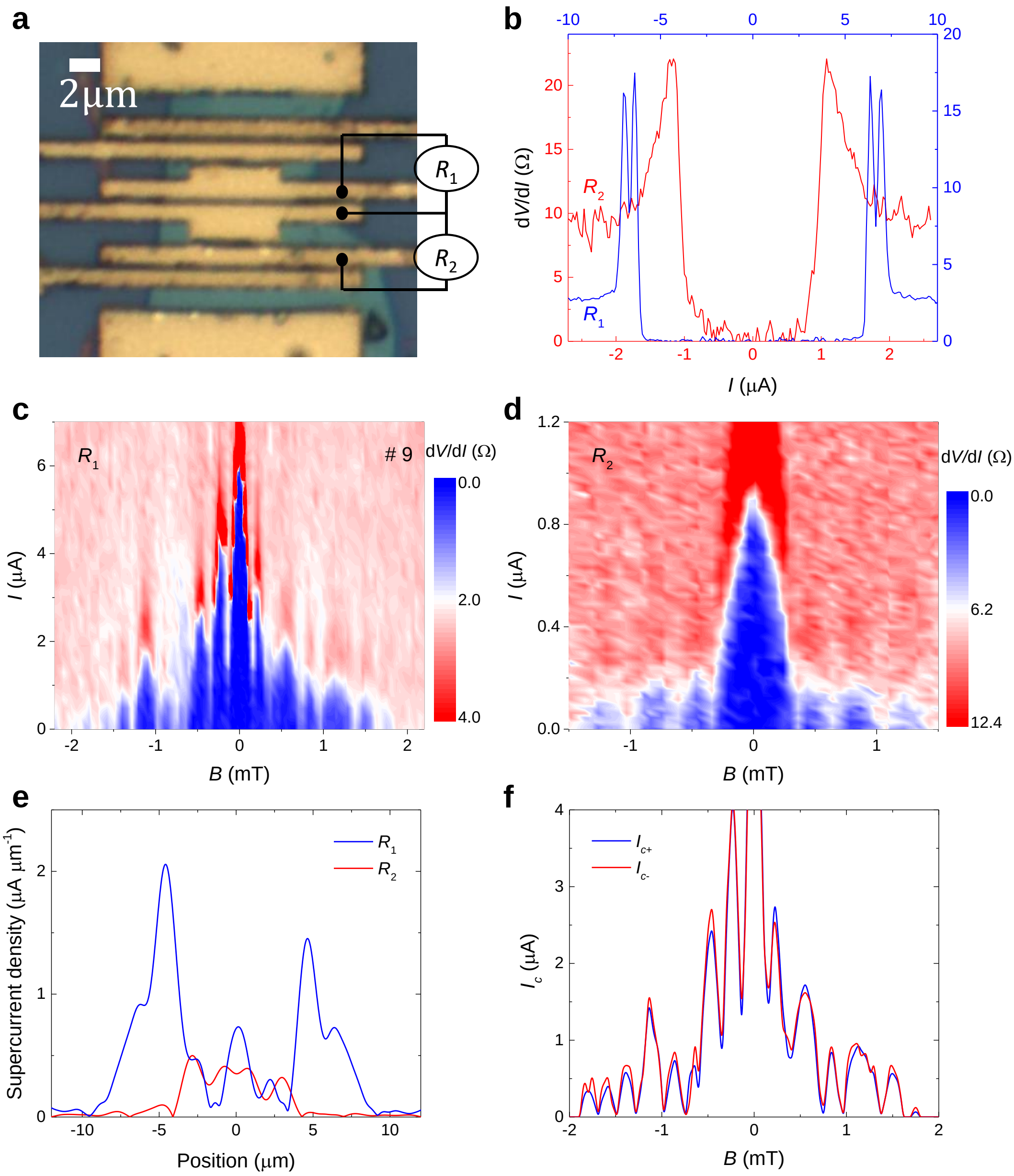

Supplementary Figure 9. Josephson junction in device # 9 (thin sample) by the two-probe method. (a) The optical image of device # 9. R_1 and R_2 represent the Josephson channel with edge region and without edge region, respectively. **(b)** The differential resistance at 0.035 K for R_1 and R_2 by blue and red curves, respectively. **(c)-(d)** SQI results for R_1 and R_2 at 0.035 K, respectively. **(e)** The supercurrent distribution for R_1 and R_2 by blue and red curves, respectively. The R_1 shows edge supercurrent while R_2 does not. **(f)** Critical current I_c for R_1 as a function of B for the two sweep directions (positive as the blue line, negative as the red line), indicating non-symmetric behavior $I_c^+(B) \neq I_c^-(B)$.

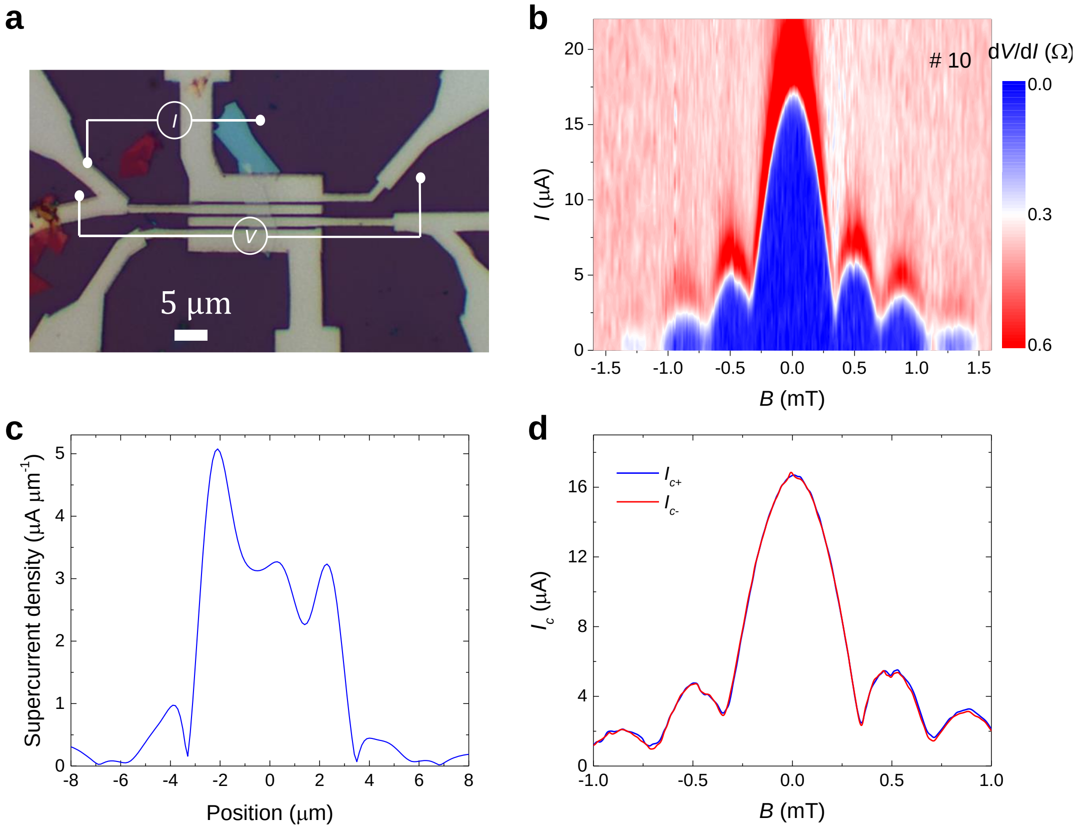

Supplementary Figure 10. Josephson junction in device # 10 (thick sample) by two-probe method at 0.035 K. (a) The optical image of device # 10 with two-probe configuration. The width and length of the channel are 6 μm and 0.3 μm, respectively. **(b)** Differential resistance under out-of-plane magnetic fields, showing a Fraunhofer pattern with a central lobe of width $2\Phi_0$. **(c)** The supercurrent distribution. **(d)** Critical current I_c as a function of B for the two sweep directions (positive as the blue line, negative as the red line), indicating the symmetric I_c relation.

VIII. Discussion on non-uniform supercurrent density

There is no doubt that thinner films are more susceptible to fluctuations and affected more by the SiO_2 substrate or the capping layer.

However, the fluctuations should be random along the in-plane direction. It is quite difficult to believe that the fluctuations are not uniform and gathered only at the edges. Moreover, four thin devices behave large edge supercurrent density while three thick devices show the Fraunhofer pattern which indicates good repeatability. Actually, we simulate the effect by random fluctuations as shown in Supplementary Fig. 11. The supercurrent density fluctuations are assumed to be $\pm 10\%$ and $\pm 30\%$ in Supplementary Fig. 11a. The $I_c - B$ relation can be estimated by

$$I_c^{max}(B) = \left| \int_{-\infty}^{\infty} J_c(x) \cos\left(\frac{2\pi L_{eff} B x}{\Phi_0}\right) \mathrm{d}x \right|$$

The result is simulated as shown in Supplementary Fig. 11b, which is a not ideal Fraunhofer pattern but almost similar to conventional unchanged supercurrent density.

The trap sites in the SiO_2 substrate or the capping layer. This effect also possibly

exists in our systems. However, such substrate or the capping layer induced non-uniform supercurrent density should affect obviously the out-of-plane direction rather than in-plane direction. Both the substrate and the capping layer can induce fluctuations or trapped carriers at the top and bottom surface of WTe_2 rather than non-uniform distribution in-plane. Therefore, this effect should be much smaller in our experiments.

Furthermore, a lot of Josephson junction works based on other thin materials have been reported before and exhibit the normal Fraunhofer pattern[7-9].

In summary, based on the three reasons listed above, we conclude that the fluctuations and effect by SiO_2 cannot result in the non-uniform supercurrent density.

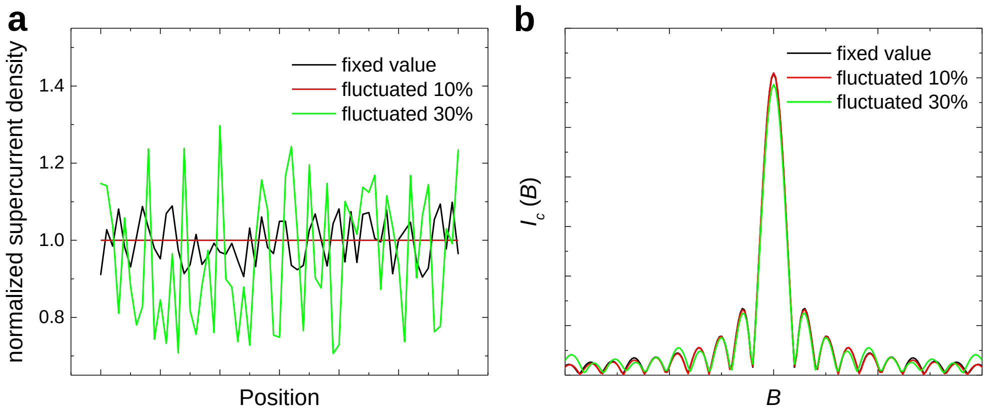

Supplementary Figure 11. Expected effect on SQI measuring by contaminations. (a) The supposed supercurrent density by fixed value and fluctuating value for no and random contamination effect, respectively. **(b)** The estimated magnetic field dependent supercurrent oscillation from **(a)** and indicates whether small fluctuating supercurrent density will almost not change the SQI results.

In graphene, “fibre-optic” modes exist at the edge due to the band bending[10]. This trivial effect will also result in the SQUID interference pattern[10]. However, this edge-mode-dominated current flow can be observed only near the Dirac point, not at a higher density of states. The conductance of thin WTe_2 is larger than the quantum conductance ($t \cdot \frac{2e^2}{h}$, t is the thickness) indicating a non-negligible bulk contribution and a high Fermi level. Thus, the edge superconductivity observed in WTe_2 does not seem to be caused by “fibre-optic” edge modes.

IX. References

1. Huang C, Narayan A, Zhang E, *et al.* Inducing Strong Superconductivity in WTe2 by a Proximity Effect. *ACS Nano*. 2018; **12**(7): 7185-96.

2. Gubin AI, Il'in KS, Vitusevich SA, *et al.* Dependence of magnetic penetration depth on the thickness of superconducting Nb thin films. *Phys Rev B*. 2005; **72**(6): 064503.

3. Borzenets IV, Amet F, Ke CT, *et al.* Ballistic Graphene Josephson Junctions from the Short to the Long Junction Regimes. *Phys Rev Lett*. 2016; **117**(23): 237002.

4. Dubos P, Courtois H, Pannetier B, *et al.* Josephson critical current in a long mesoscopic S-N-S junction. *Phys Rev B*. 2001; **63**(6): 064502.

5. Dynes RC, Fulton TA. Supercurrent Density Distribution in Josephson Junctions. *Phys Rev B*. 1971; **3**(9): 3015-23.

6. Lee JH, Lee G-H, Park J, *et al.* Local and Nonlocal Fraunhofer-like Pattern from an Edge-Stepped Topological Surface Josephson Current Distribution. *Nano Lett*. 2014; **14**(9): 5029-34.

7. Heersche HB, Jarillo-Herrero P, Oostinga JB, *et al.* Bipolar supercurrent in graphene. *Nature*. 2007; **446**(7131): 56-9.

8. Du X, Skachko I, Andrei EY. Josephson current and multiple Andreev reflections in graphene SNS junctions. *Phys Rev B*. 2008; **77**(18): 184507.

9. Cho S, Dellabetta B, Yang A, *et al.* Symmetry protected Josephson supercurrents in three-dimensional topological insulators. *Nat Commun*. 2013; **4**(1): 1689.

10. Allen MT, Shtanko O, Fulga IC, *et al.* Spatially resolved edge currents and guided-wave electronic states in graphene. *Nat Phys*. 2015; **12**: 128.
